# Supplementary material for: Body mass index and risk of dying from a bloodstream infection: A Mendelian randomization study
Source: PLoS Med. 2020 Nov 16;17(11):e1003413. doi: 10.1371/journal.pmed.1003413 (PMC7668585; doi:10.1371/journal.pmed.1003413)
Supplement: S1 Checklist — (PDF) [file pmed.1003413.s001.pdf]

PeerJ Preprints NOT PEER-REVIEWED

STROBE-MR: Guidelines for strengthening the reporting of Mendelian randomization studies

While the number of studies using Mendelian randomization (MR) methods has grown exponentially in the last decade, the quality of reporting of these studies often has been poor. Similar to other reporting guidelines such as CONSORT (*Consolidated Standards of Reporting Trials*) for randomised trials and STROBE (*STrenghtening the Reporting of Observational studies in Epidemiology*) for observational studies in epidemiology, the STROBE-MR working group aims to provide guidance to authors on how to improve reporting of MR studies and help readers, reviewers, and journal editors to evaluate the quality of the presented evidence.

Empirical evidence indicates that many reports of MR studies do not clearly state or examine the various assumptions of MR methods and report insufficient details on the data sources, which makes it hard to evaluate the quality and reliability of the results. The STROBE-MR guidance covers both one sample and two sample MR studies. At present, the draft checklist consists of 20 items, organized into the title and abstract, introduction, methods, results and discussion sections of articles.

As these guidelines aim to reach the entire MR community, we would like to give everyone the opportunity to contribute their comments. The following draft of the STROBE-MR checklist is open for public discussion and all feedback will be taken into account during its next revision. For feedback, please use the comment section below this post on PeerJ Preprints.

We hope the final guidelines will serve the entire community and contribute to improving the reporting of MR studies in the future.

PeerJ Preprints | https://doi.org/10.7287/peerj.preprints.27857v1 | CC BY 4.0 Open Access | rec: 15 Jul 2019, publ: 15 Jul 2019

PeerJ Preprints NOT PEER-REVIEWED

- DRAFT -

STROBE-MR: Guidelines for strengthening the reporting of Mendelian randomization studies

Authors (in alphabetical order):

George Davey Smith, Neil M Davies, Niki Dimou, Matthias Egger, Valentina Gallo, Robert Golub, Julian PT Higgins, Claudia Langenberg, Elizabeth W Loder, J Brent Richards, Rebecca C Richmond, Veronika W Skrivankova, Sonja A Swanson, Nicholas J Timpson, Anne Tybjaerg-Hansen, Tyler J VanderWeele, Benjamin AR Woolf, James Yarmolinsky

Corresponding author: Veronika W Skrivankova
veronika.skrivankova@ispm.unibe.ch

Introduction

While the number of studies using Mendelian randomization (MR) methods has grown exponentially in the last decade, the quality of reporting of these studies often has been poor (*Davies et al, 2013; Swanson & Hernán, 2013; Davies, Holmes & Davey Smith, 2018*). Similar to other reporting guidelines such as CONSORT (*Altman et al, 2001; Moher et al, 2001*) for randomised trials and STROBE (*von Elm et al, 2007; Vandenbroucke et al, 2007*) for observational studies in epidemiology, the STROBE-MR working group aims to provide guidance to authors on how to improve reporting of MR studies and help readers, reviewers, and journal editors to evaluate the quality of the presented evidence.

Empirical evidence indicates that many reports of MR studies do not clearly state or examine the various assumptions of MR methods and report insufficient details on the data sources, which makes it hard to evaluate the quality and reliability of the results. The STROBE-MR guidance covers both one sample and two sample MR studies. At present, the draft checklist consists of 20 items, organized into the title and abstract, introduction, methods, results and discussion sections of articles.

As these guidelines aim to reach the entire MR community, we would like to give everyone the opportunity to contribute their comments. The following draft of the STROBE-MR checklist is open for public discussion and all feedback will be taken into account during its next revision. For feedback, please use the comment section below this post on PeerJ Preprints.

We hope the final guidelines will serve the entire community and contribute to improving the reporting of MR studies in the future.

PeerJ Preprints | https://doi.org/10.7287/peerj.preprints.27857v1 | CC BY 4.0 Open Access | rec: 15 Jul 2019, publ: 15 Jul 2019

PeerJ Preprints NOT PEER-REVIEWED

Title and abstract

1. TITLE and ABSTRACT

Indicate Mendelian randomization as the study's design in the title and/or the abstract.

INTRODUCTION

Introduction, paragraphs 1-4

2. Background

Explain the scientific background and rationale for the reported study. Is causality between exposure and outcome plausible? Justify why MR is a helpful method to address the study question.

Introduction, paragraph 4

3. Objectives

State specific objectives clearly, including pre-specified causal hypotheses (if any).

METHODS

4. Study design and data sources

Present key elements of study design early in the paper. Consider including a table listing sources of data for all phases of the study. For each data source contributing to the analysis, describe the following:

Methods, paragraphs 1-5
a) Describe the study design and the underlying population from which it was drawn. Describe also the setting, locations, and relevant dates, including periods of recruitment, exposure, follow-up, and data collection, if available.

Methods, paragraph 8
b) Give the eligibility criteria, and the sources and methods of selection of participants.

Methods, paragraph 8
c) Explain how the analyzed sample size was arrived at.

Methods, paragraph 7
d) Describe measurement, quality and selection of genetic variants.

Methods, paragraphs 2 and 4
e) For each exposure, outcome and other relevant variables, describe methods of assessment and, in the case of diseases, the diagnostic criteria used.

Methods; Ethical approval, paragraph 1
f) Provide details of ethics committee approval and participant informed consent, if relevant.

Methods; Statistical analysis, paragraph 1,3,4

5. Assumptions

Explicitly state assumptions for the main analysis (e.g. relevance, exclusion, independence, homogeneity) as well assumptions for any additional or sensitivity analysis.

6. Statistical methods: main analysis

Describe statistical methods and statistics used.

Methods; Statistical analysis, paragraph 1,3,4
a) Describe how quantitative variables were handled in the analyses (i.e., scale, units, model).

PeerJ Preprints | https://doi.org/10.7287/peerj.preprints.27857v1 | CC BY 4.0 Open Access | rec: 15 Jul 2019, publ: 15 Jul 2019

PeerJ Preprints NOT PEER-REVIEWED

Methods, paragraph 7

b) Describe the process for identifying genetic variants and weights to be included in the analyses (i.e, independence and model). Consider a flow diagram.

Methods; Statistical analysis, paragraph 3,4 S1 Text

c) Describe the MR estimator, e.g. two-stage least squares, Wald ratio, and related statistics. Detail the included covariates and, in case of two-sample MR, whether the same covariate set was used for adjustment in the two samples.

d) Explain how missing data were addressed.

e) If applicable, say how multiple testing was dealt with.

7. Assessment of assumptions

Methods; Statistical analysis, paragraph 4,5

Describe any methods used to assess the assumptions or justify their validity.

8. Sensitivity analyses

Methods; Statistical analysis, paragraph 3-6

Describe any sensitivity analyses or additional analyses performed.

9. Software and pre-registration

Methods; Statistical analysis, paragraph 7

a) Name statistical software and package(s), including version and settings used.

Methods, paragraph 9

b) State whether the study protocol and details were pre-registered (as well as when and where).

RESULTS

10. Descriptive data

Results, paragraph 1

a) Report the numbers of individuals at each stage of included studies and reasons for exclusion. Consider use of a flow-diagram.

Results, paragraph 1, Table 1

b) Report summary statistics for phenotypic exposure(s), outcome(s) and other relevant variables (e.g. means, standard deviations, proportions).

N/A

c) If the data sources include meta-analyses of previous studies, provide the number of studies, their reported ancestry, if available, and assessments of heterogeneity across these studies. Consider using a supplementary table for each data source.

Results; Sensitivity analyses, paragraph 4

d) For two-sample Mendelian randomization:

i. Provide information on the similarity of the genetic variant-exposure associations between the exposure and outcome samples.

ii. Provide information on extent of sample overlap between the exposure and outcome data sources.

PeerJ Preprints NOT PEER-REVIEWED

11. Main results

Results, paragraph 2	a) Report the associations between genetic variant and exposure, and between genetic variant and outcome, preferably on an interpretable scale (e.g. comparing 25th and 75th percentile of allele count or genetic risk score, if individual-level data available).
Results, paragraph 2,3	b) Report causal effect estimate between exposure and outcome, and the measures of uncertainty from the MR analysis. Use an intuitive scale, such as odds ratio, or relative risk, per standard deviation difference.
	c) If relevant, consider translating estimates of relative risk into absolute risk for a meaningful time-period.
Fig 1-3	d) Consider any plots to visualize results (e.g. forest plot, scatterplot of associations between genetic variants and outcome versus between genetic variants and exposure).

12. Assessment of assumptions

Results; Sensitivity analysis, paragraph 2-5	a) Assess the validity of the assumptions.
	b) Report any additional statistics (e.g., assessments of heterogeneity, such as I^2, Q statistic).

13. Sensitivity and additional analyses

Results; Sensitivity analysis, paragraph 1-6	a) Use sensitivity analyses to assess the robustness of the main results to violations of the assumptions.
Results; Sensitivity analysis, paragraph 1-6	b) Report results from other sensitivity analyses (e.g., replication study with different dataset, analyses of subgroups, validation of instrument(s), simulations, etc.).
	c) Report any assessment of direction of causality (e.g., bidirectional MR).
Results; Sensitivity analysis, paragraph 1	d) When relevant, report and compare with estimates from non-MR analyses.
S1 Fig - S7 Fig	e) Consider any additional plots to visualize results (e.g., leave-one-out analyses).

DISCUSSION

14. Key results

Discussion, paragraph 1	Summarize key results with reference to study objectives.

15. Limitations

Discussion; Strengths and limitations paragraph 1	Discuss limitations of the study, taking into account the validity of the MR assumptions, other sources of potential bias, and imprecision. Discuss both direction and magnitude of any potential bias, and any efforts to address them.

PeerJ Preprints | https://doi.org/10.7287/peerj.preprints.27857v1 | CC BY 4.0 Open Access | rec: 15 Jul 2019, publ: 15 Jul 2019

PeerJ Preprints NOT PEER-REVIEWED

16. Interpretation

Discussion; paragraphs 2-6

a) Give a cautious overall interpretation of results considering objectives and limitations. Compare with results from other relevant studies.

b) Discuss underlying biological mechanisms that could be modelled by using the genetic variants to assess the relationship between the exposure and the outcome.

Discussion; Conclusion, paragraph 1

c) Discuss whether the results have clinical or policy relevance, and whether interventions could have the same size effect.

17. Generalizability

Discussion; Strengths and limitations paragraph 1

Discuss the generalizability of the study results (a) to other populations (i.e. external validity), (b) across other exposure periods/timings, and (c) across other levels of exposure.

OTHER INFORMATION

18. Funding

Meta-data

Give the source of funding and the role of the funders for the present study and, if applicable, for the original study or studies on which the present article is based.

Results; Data statement, paragraphs 1-2

19. Data and data sharing

Present data used to perform all analyses or report where and how the data can be accessed. State whether statistical code is publicly accessible and if so, where.

Meta-data

20. Conflicts of Interest

All authors should declare all potential conflicts of interest.

References:

Altman, D. G., Schulz, K. F., Moher, D., Egger, M., Davidoff, F., Elbourne, D., and Lang, T. (2001). The Revised CONSORT Statement for Reporting Randomized Trials: Explanation and Elaboration. *Ann of Internal Med*, *134*(8), 663–694.

Davies, N. M., Davey Smith, G., Windmeijer, F., and Martin, R. M. (2013). Issues in the Reporting and Conduct of Instrumental Variable Studies A Systematic Review. *Epidemiology*, *24*(3), 363–369.

Davies, N. M., Holmes, M. V, and Davey Smith, G. (2018). Reading Mendelian randomisation studies: a guide, glossary, and checklist for clinicians. *BMJ*, *362:*k601.

Moher, D., Schulz, K. F., Altman, D. G., and Lepage, L. (2001). The CONSORT Statement: Revised Recommendations for Improving the Quality of Reports of Parallel-Group Randomised Trials. *Lancet*, *357*(9263), 1191–1194.

Swanson, S. A., and Hernán, M. A. (2013). How to Report Instrumental Variable Analyses. *Epidemiology*, *24*(3).

Von Elm, E., Altman, D. G., Egger, M., Pocock, S. J., Gøtzsche, P. C., Vandenbroucke, J. P., and the STROBE initiative (2007). The Strengthening the Reporting of Observational Studies in Epidemiology (STROBE) Statement: Guidelines for Reporting Observational Studies. *Lancet*, *370*(9596)*,* 1453–7.

Vandenbroucke, J. P., von Elm, E., Altman, D. G., Gøtzsche, P. C., Mulrow, C. D., Pocock, S. J., Poole, C., Schlesselman, J. J., and Egger, M. (2007). Strengthening the Reporting of Observational Studies in Epidemiology (STROBE): Explanation and Elaboration. *PLoS Medicine*, *4*(10), e297.
