# Supplementary figures and images for: Body mass index and risk of dying from a bloodstream infection: A Mendelian randomization study
Source: PLoS Med. 2020 Nov 16;17(11):e1003413. doi: 10.1371/journal.pmed.1003413 (PMC7668585; doi:10.1371/journal.pmed.1003413)

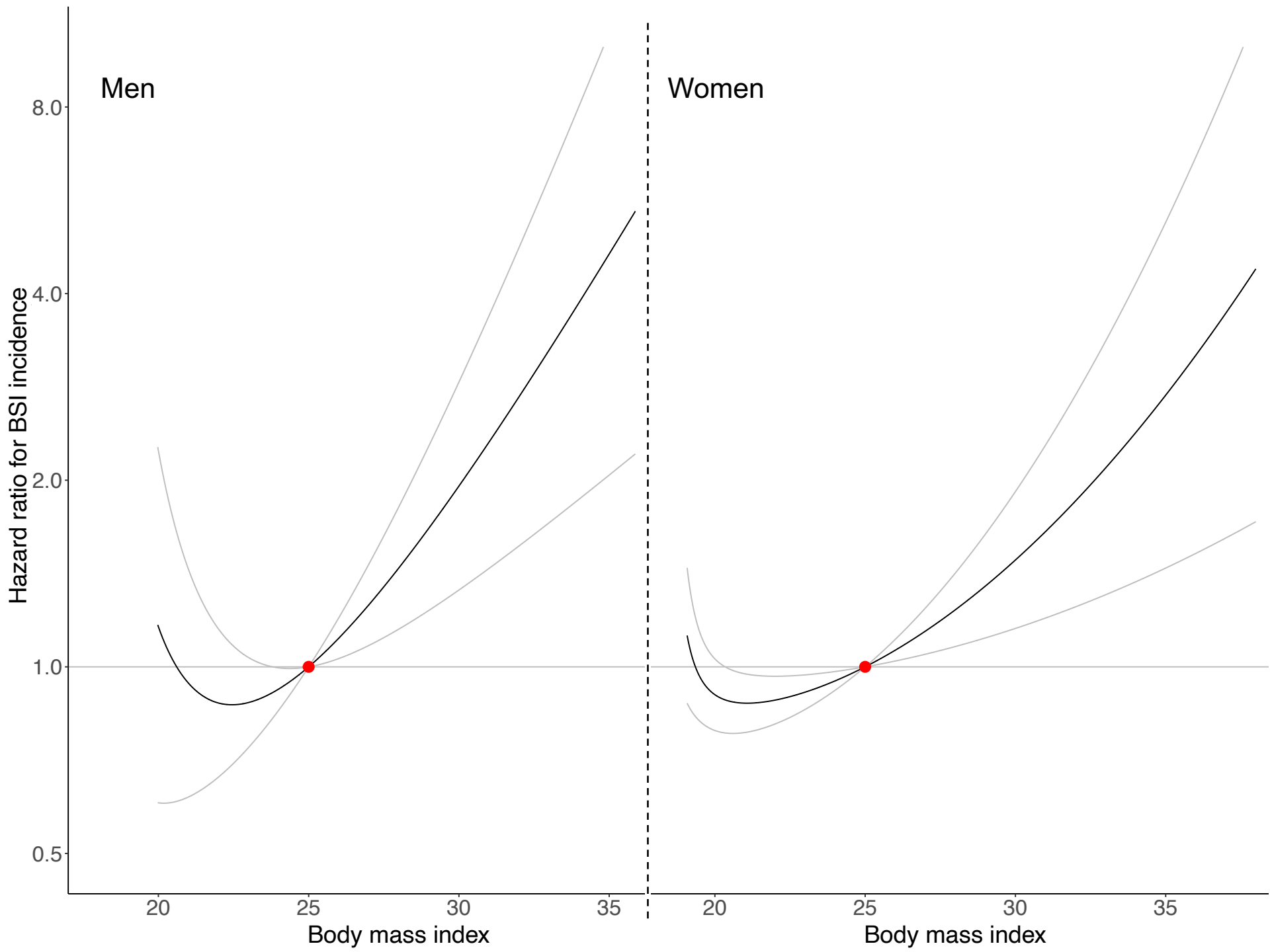

Supplement: S1 Fig — The association between genetically predicted body mass index and risk of contracting a bloodstream infection among men (left panel) and women (right panel), with a body mass index of 25 kg/m2 as reference. Gray lines represent 95% confidence intervals. (PDF) [file pmed.1003413.s003.pdf]

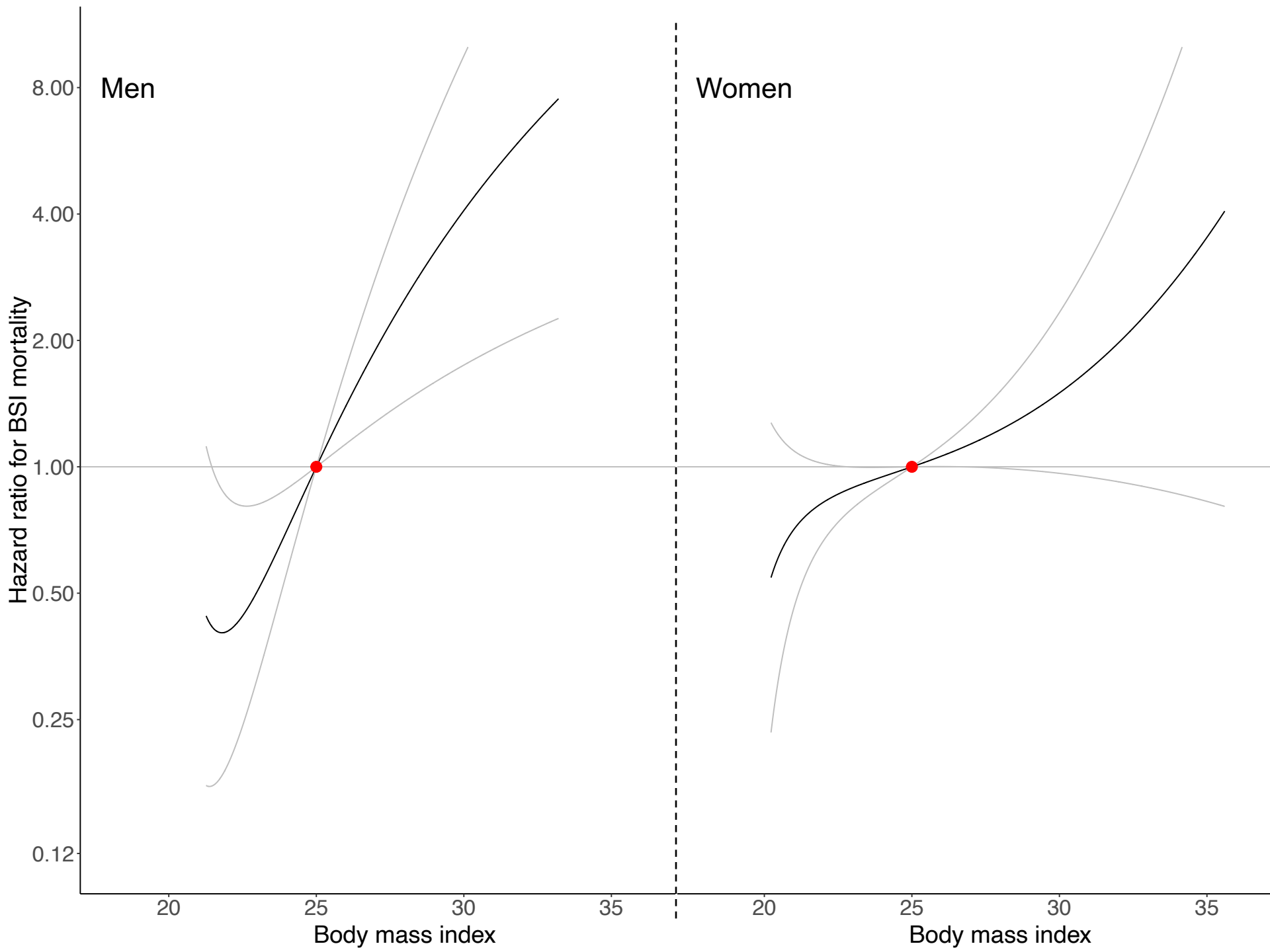

Supplement: S2 Fig — The association between genetically predicted body mass index and risk of dying from a bloodstream infection among men (left panel) and women (right panel) in the general population, with a body mass index of 25 kg/m2 as reference. Gray lines represent 95% confidence intervals. (PDF) [file pmed.1003413.s004.pdf]

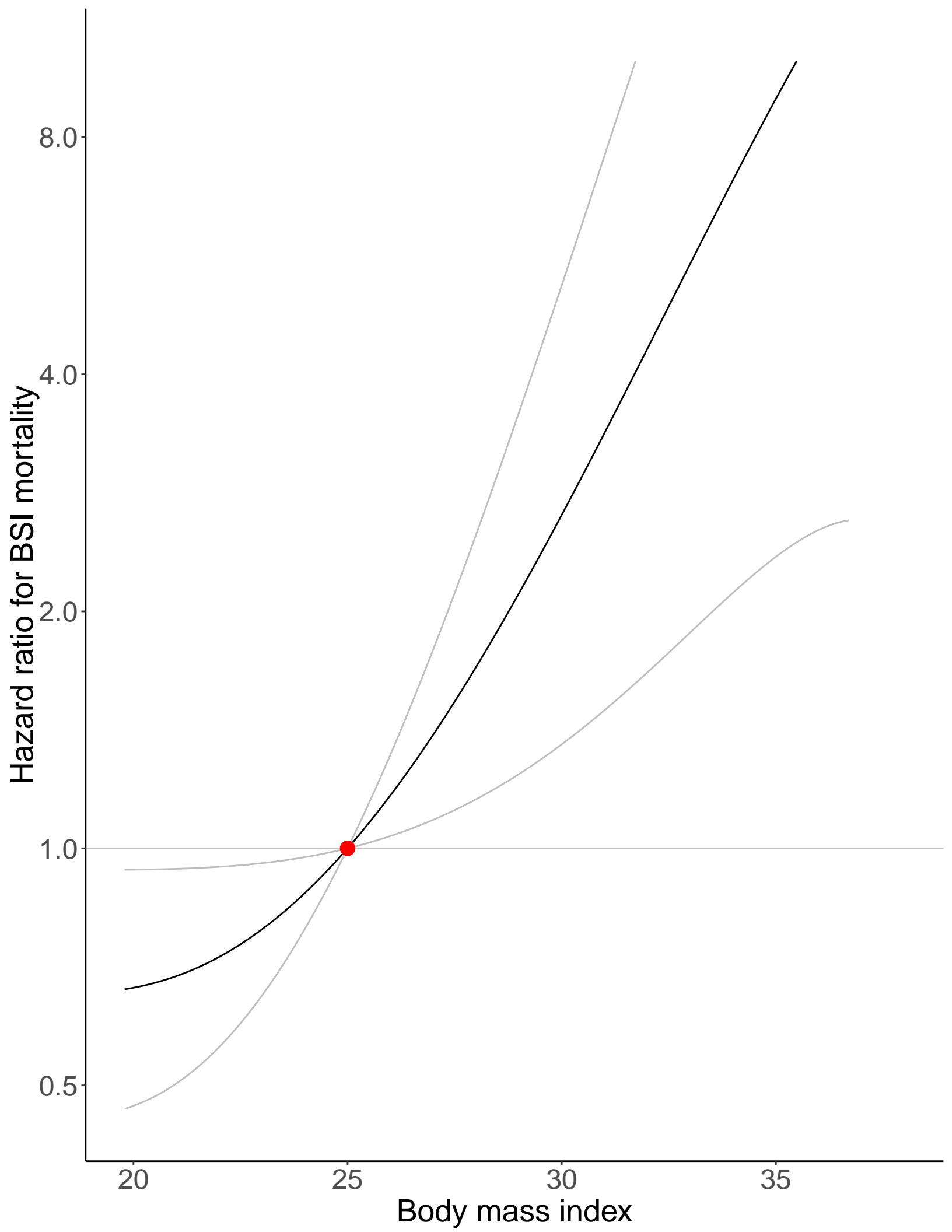

Supplement: S3 Fig — The association between genetically predicted body mass index and risk of dying from a bloodstream infection in the general population, with a body mass index of 25 kg/m2 as reference, adjusting for 5 principal components. Gray lines represent 95% confidence intervals. (PDF) [file pmed.1003413.s005.pdf]

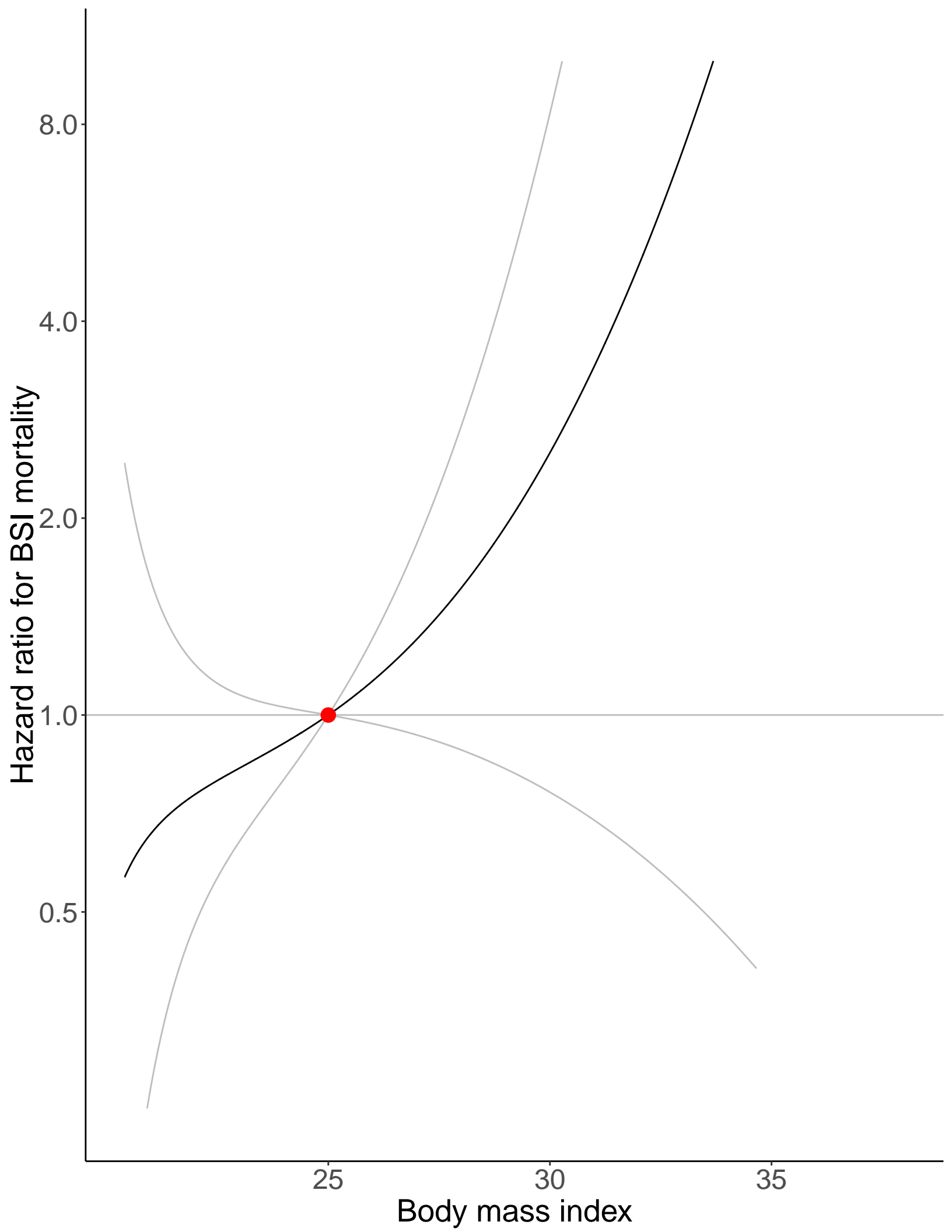

Supplement: S4 Fig — The association between genetically predicted body mass index and risk of dying from a bloodstream infection in the general population, with a body mass index of 25 kg/m2 as reference. Gray lines represent 95% confidence intervals. Single nucleotide polymorphisms used were rs11075986, rs16952479, rs2075205, rs3751813, rs8047395, rs9922708, and rs9931164. (PDF) [file pmed.1003413.s006.pdf]

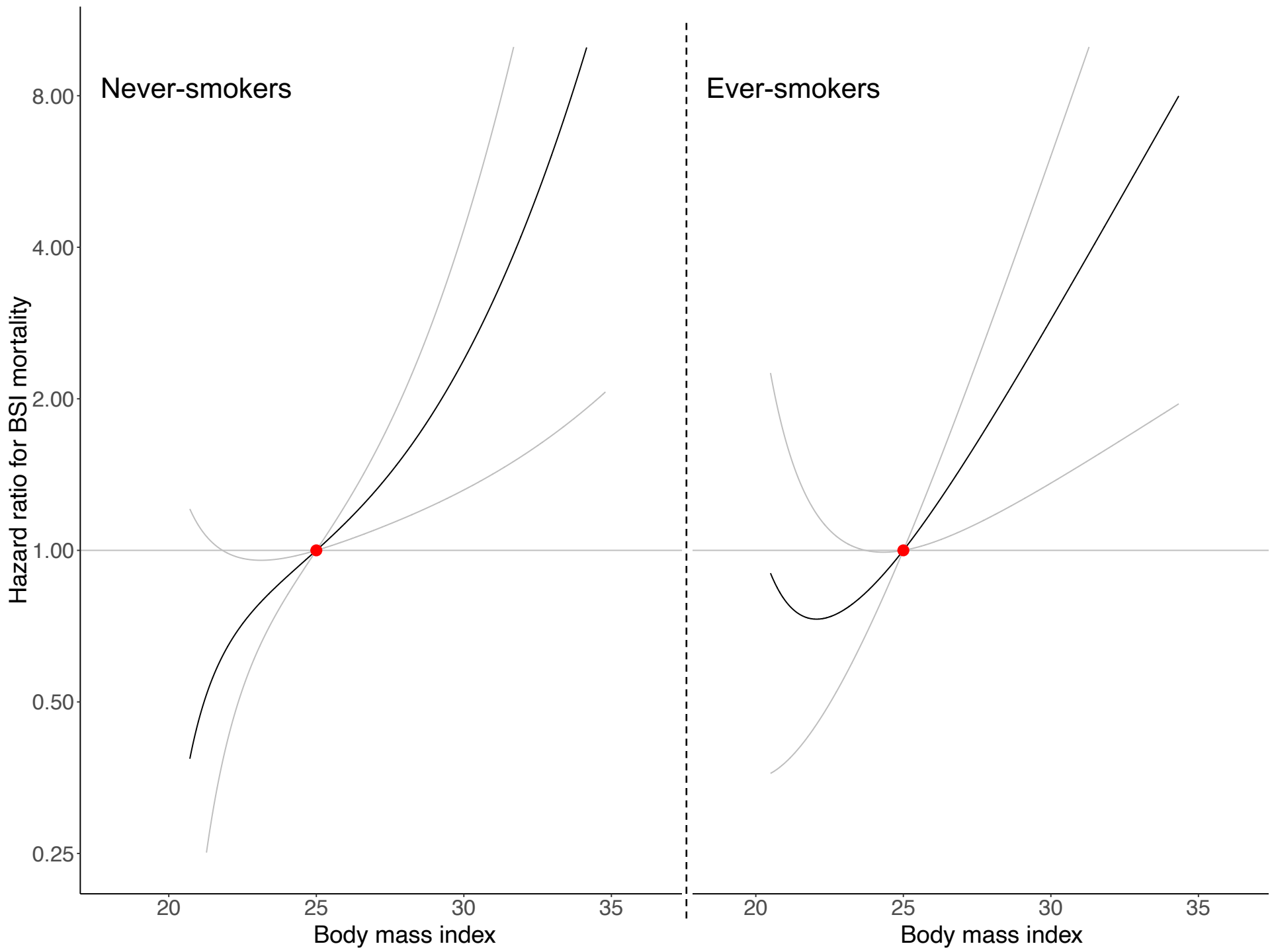

Supplement: S5 Fig — The association between genetically predicted body mass index and risk of dying from a bloodstream infection among never-smokers (left panel) and ever-smokers (right panel) in the general population, with a body mass index of 25 kg/m2 as reference. Gray lines represent 95% confidence intervals. (PDF) [file pmed.1003413.s007.pdf]

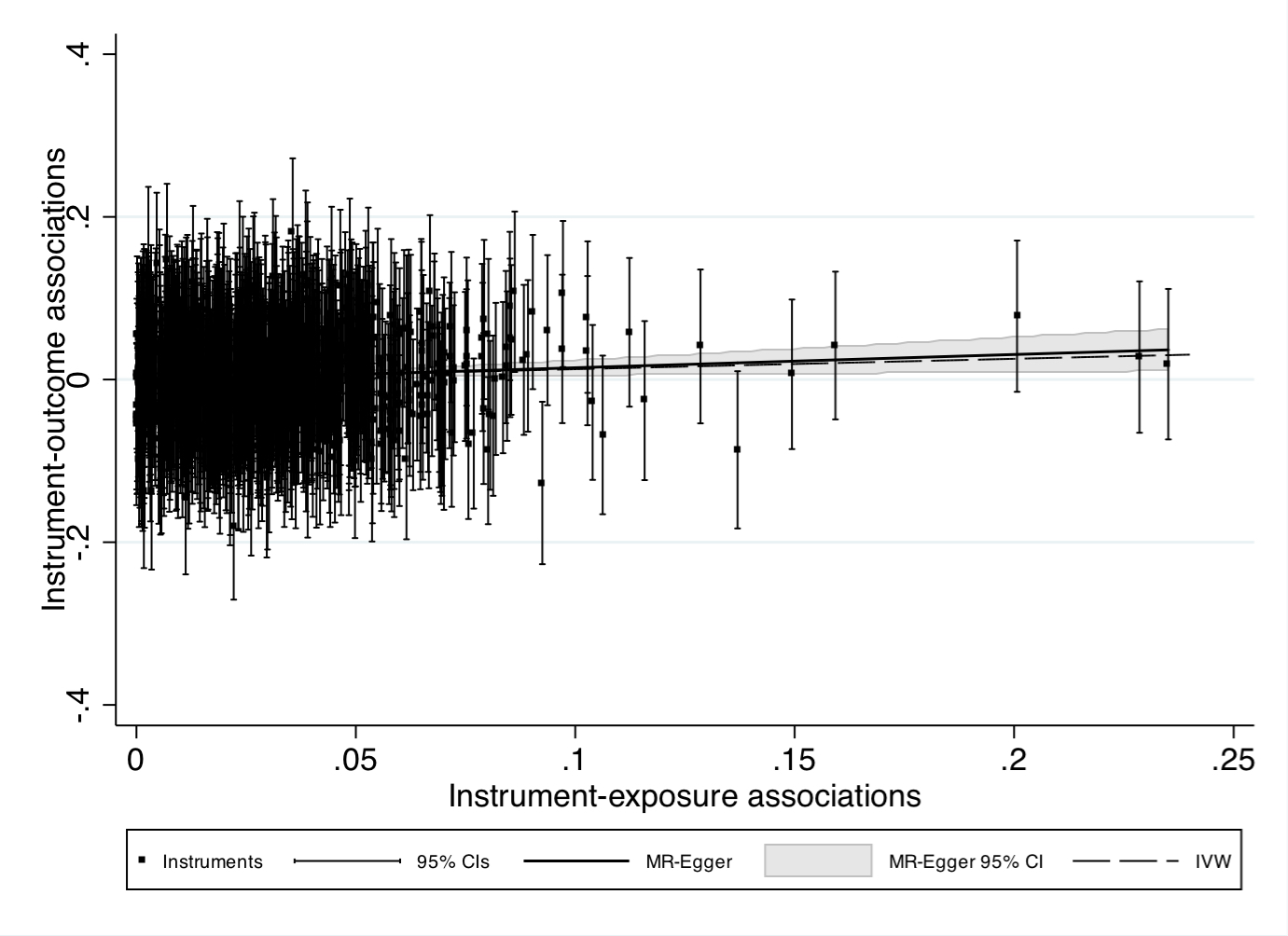

Supplement: S6 Fig — Single nucleotide polymorphism–body mass index association and single nucleotide polymorphism–bloodstream infection mortality association in the general population, with inverse-variance-weighted regression for comparison. (JPG) [file pmed.1003413.s008.jpg]

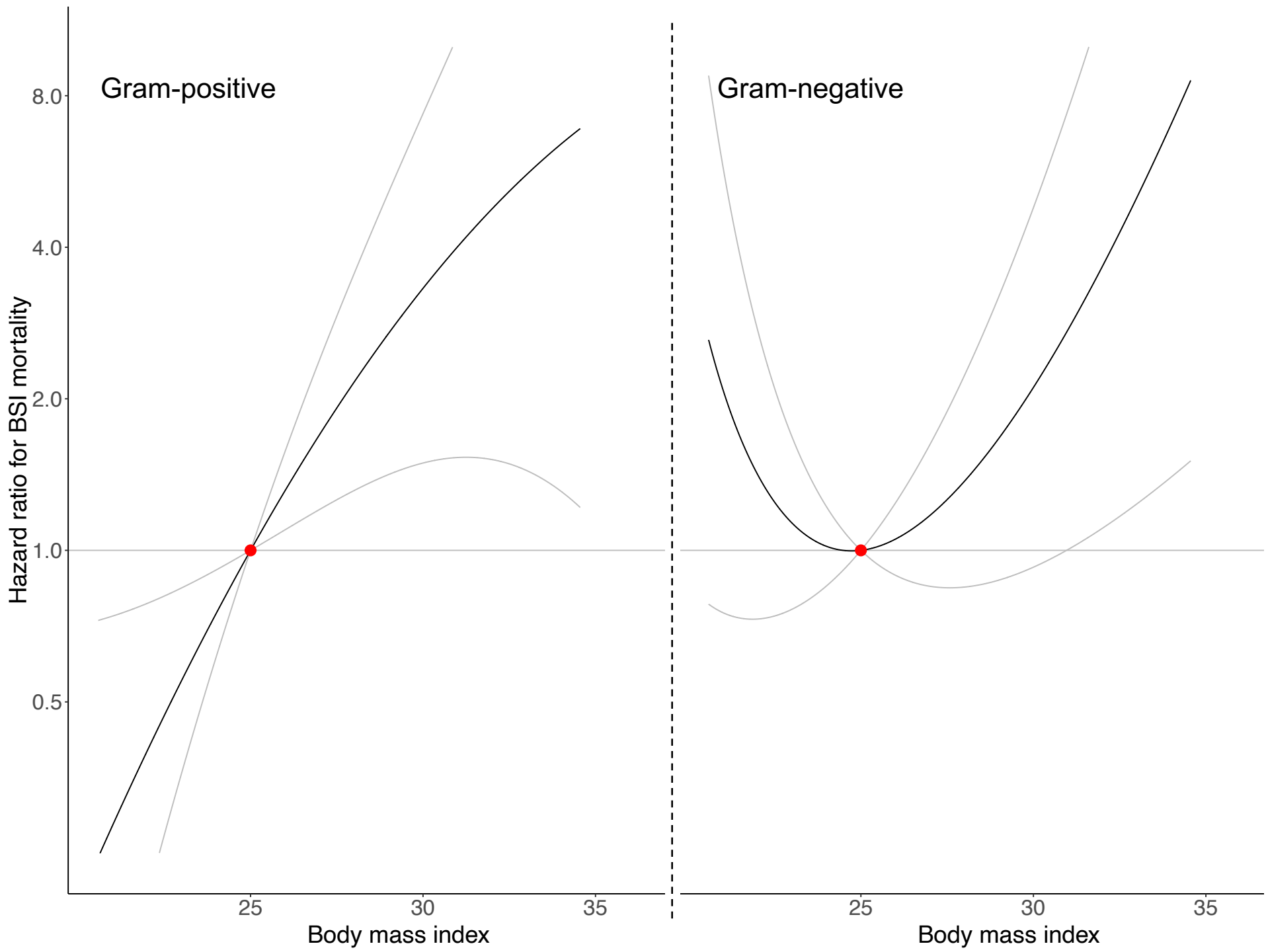

Supplement: S7 Fig — The association between genetically predicted body mass index and risk of dying from a bloodstream infection due to gram-positive (left panel) and gram-negative bacteria (right panel), with a body mass index of 25 kg/m2 as reference. Gray lines represent 95% confidence intervals. There were 220 deaths due to gram-positive bloodstream infection and 215 deaths due to gram-negative bloodstream infection. (PDF) [file pmed.1003413.s009.pdf]
