# Supplementary material for: Body mass index and risk of dying from a bloodstream infection: A Mendelian randomization study
Source: PLoS Med. 2020 Nov 16;17(11):e1003413. doi: 10.1371/journal.pmed.1003413 (PMC7668585; doi:10.1371/journal.pmed.1003413)
Supplement: S1 Table — BMI, body mass index; BSI, bloodstream infection; HR, hazard ratio. The 6 strata correspond to the BMI categories underweight, normal weight, overweight, obese class I, obese class II, and obese class III (from the top). The BMI strata were created based on residual BMI and observed BMI in the Mendelian randomization and multivariable analysis, respectively. The HR corresponds to a 1-kg/m2 increase in BMI within that stratum. (DOCX) [file pmed.1003413.s010.docx]

| **S1 Table. Two-stage least squares Mendelian randomization vs multivariable Cox regression of body mass index and bloodstream infection incidence** | | | | | | | | | | | |
| --- | --- | --- | --- | --- | --- | --- | --- | --- | --- | --- | --- |
|  | **Mendelian randomization**  **(n = 55,908)** | | | | |  | **Standard analysis: Adjusting for age, sex,**  **education, smoking, activity and cancer**  **(n = 47,201)** | | | | |
| **BMI** | **Person-years at risk** | **No. BSI  events** | **Incidence rate per 100,000 person-years** | **HR** | **95% CI** |  | **Person-years at risk** | **No. BSI  events** | **Incidence rate per 100,000 person-years** | **HR** | **95% CI** |
| Overall | 993,912 | 2,547 | 256 | 1.10 | 1.05-1.16 |  | 866,346 | 1,853 | 214 | 1.06 | 1.04-1.07 |
| <18.5 | 6,148 | 11 | 179 | 0.44 | 0.16-1.21 |  | 5,104 | 8 | 157 | 0.47 | 0.18-1.27 |
| 18.5-24.9 | 396,550 | 684 | 172 | 1.03 | 0.93-1.15 |  | 355,166 | 516 | 145 | 0.94 | 0.89-1.00 |
| 25.0-29.9 | 439,368 | 1224 | 279 | 1.16 | 1.04-1.19 |  | 378,740 | 878 | 232 | 1.09 | 1.04-1.14 |
| 30.0-34.9 | 123,107 | 471 | 383 | 1.14 | 1.04-1.26 |  | 102,333 | 334 | 326 | 1.15 | 1.06-1.24 |
| 34.9-39.9 | 23,340 | 117 | 501 | 1.14 | 0.94-1.38 |  | 19,940 | 89 | 446 | 0.99 | 0.85-1.16 |
| >40.0 | 5,399 | 40 | 741 | 1.10 | 0.81-1.50 |  | 5,063 | 28 | 553 | 0.98 | 0.84-1.14 |
| BMI, body mass index; BSI, bloodstream infection; HR, hazard ratio. The six strata correspond to the BMI categories underweight, normal weight, overweight, obese class I, obese class II, and obese class III (from the top). BMI strata created based on residual BMI and observed BMI in the Mendelian randomization and multivariable analysis, respectively. The HR corresponds to a 1 kg/m^2^ increase in BMI within that stratum. | | | | | | | | | | | |
