# Supplementary material for: Body mass index and risk of dying from a bloodstream infection: A Mendelian randomization study
Source: PLoS Med. 2020 Nov 16;17(11):e1003413. doi: 10.1371/journal.pmed.1003413 (PMC7668585; doi:10.1371/journal.pmed.1003413)
Supplement: S3 Table — BMI, body mass index; Q, quartile; SD, standard deviation. Post-secondary defined as at least some university or other post-secondary education. Moderate/high activity defined as ≥3 h light activity/week or any vigorous activity/week. (DOCX) [file pmed.1003413.s012.docx]

| **S3 Table. Distribution of potential confounders by body mass index quartiles** | | | | | | | | | | |
| --- | --- | --- | --- | --- | --- | --- | --- | --- | --- | --- |
|  | **Quartiles of BMI** | | | | | | | | | |
|  | **Q1** | | **Q2** | | **Q3** | | **Q4** | | **Test for trend** | |
| Quartile range, kg/m^2^ | 14.8 to 23.5 | | 23.6 to 25.8 | | 25.9 to 28.6 | | 28.7 to 53.3 | |  |  |
|  |  |  |  |  |  |  |  |  |  |  |
|  | *n* | *%* | *n* | *%* | *n* | *%* | *n* | *%* | *R^2^ %* | *P-value* |
| Male sex | 4,954 | 35.4 | 7,252 | 51.8 | 8,051 | 56.3 | 6,068 | 44.5 | 0.4 | <0.001 |
| Self-reported cancer | 439 | 3.3 | 464 | 3.5 | 480 | 3.5 | 572 | 4.5 | 0.2 | <0.001 |
| Never smoked | 5,854 | 42.7 | 5,873 | 42.7 | 5,965 | 42.5 | 5,903 | 44.3 | <0.1 | 0.014 |
| Current smoker | 5,205 | 38.0 | 4,181 | 30.4 | 3,666 | 26.1 | 3,065 | 23.0 | 1.2 | <0.001 |
| Post-secondary education | 3,375 | 25.0 | 2,993 | 22.2 | 2,649 | 19.5 | 1,817 | 14.2 | 0.9 | <0.001 |
| Moderate/high activity | 8,424 | 65.1 | 8,476 | 66.2 | 7,879 | 61.1 | 6,199 | 52.5 | 0.7 | <0.001 |
|  |  |  |  |  |  |  |  |  |  |  |
|  | *Mean* | *SD* | *Mean* | *SD* | *Mean* | *SD* | *Mean* | *SD* |  |  |
| Age (years) | 44.5 | 16.8 | 48.6 | 16.3 | 51.4 | 15.9 | 53.7 | 16.0 | 4.2 | <0.001 |
| BMI (kg/m^2^) | 21.8 | 1.4 | 24.7 | 0.7 | 27.1 | 0.8 | 31.9 | 3.1 | 79.3 | <0.001 |
| BMI, body mass index; Q, quartile; SD, standard deviation. Post-secondary defined as at least “university or other post-secondary education, less than 4 years”. Moderate/high activity defined as at least “≥3 h light activity/week or <1 h vigorous activity/week”. | | | | | | | | | | |
|  |  |  |  |  |  |  |  |  |  |  |
