# Supplementary material for: Body mass index and risk of dying from a bloodstream infection: A Mendelian randomization study
Source: PLoS Med. 2020 Nov 16;17(11):e1003413. doi: 10.1371/journal.pmed.1003413 (PMC7668585; doi:10.1371/journal.pmed.1003413)
Supplement: S4 Table — BMI, body mass index; GRS, genetic risk score; Q, quartile; SD, standard deviation. Post-secondary defined as at least some university or other post-secondary education. Moderate/high activity defined as ≥3 h light activity/week or any vigorous activity/week. (DOCX) [file pmed.1003413.s013.docx]

| **S4 Table. Distribution of potential confounders by genetic risk score for body mass index quartiles** | | | | | | | | | | |
| --- | --- | --- | --- | --- | --- | --- | --- | --- | --- | --- |
|  | **Quartiles of GRS for BMI** | | | | | | | | | |
|  | **Q1** | | **Q2** | | **Q3** | | **Q4** | | **Test for trend** | |
| Quartile range | -1.2E-3 to -9.0E-4 | | -9.0E-4 to -7.8E-4 | | -7.8E-4 to -6.7E-4 | | -6.7E-4 to 8.6E-6 | |  |  |
|  |  |  |  |  |  |  |  |  |  |  |
|  | *n* | *%* | *n* | *%* | *n* | *%* | *n* | *%* | *R^2^ %* | *P-value* |
| Male sex | 6,721 | 48.1 | 6,586 | 47.1 | 6,528 | 46.7 | 6,490 | 46.5 | <0.1 | 0.005 |
| Self-reported cancer | 506 | 3.8 | 480 | 3.6 | 473 | 3.6 | 496 | 3.7 | <0.1 | 0.739 |
| Never smoked | 6,196 | 45.2 | 6,073 | 44.2 | 5,791 | 42.2 | 5,535 | 40.5 | <0.1 | <0.001 |
| Current smoker | 3,688 | 26.9 | 3,952 | 28.8 | 4,181 | 30.5 | 4,296 | 31.4 | 0.1 | <0.001 |
| Post-secondary education | 2,882 | 21.6 | 2,782 | 20.9 | 2,581 | 19.4 | 2,589 | 19.5 | <0.1 | <0.001 |
| Moderate/high activity | 7,857 | 61.9 | 7,788 | 62.0 | 7,716 | 61.0 | 7,617 | 60.7 | <0.1 | 0.016 |
|  |  |  |  |  |  |  |  |  |  |  |
|  | Mean | SD | Mean | SD | Mean | SD | Mean | SD |  |  |
| Age (years) | 49.8 | 16.6 | 49.6 | 16.6 | 49.3 | 16.6 | 49.4 | 16.5 | <0.1 | 0.010 |
| BMI (kg/m^2^) | 25.4 | 3.7 | 26.0 | 3.8 | 26.5 | 4.1 | 27.5 | 4.4 | 3.4 | <0.001 |
| BMI, body mass index; GRS, genetic risk score; Q, quartile; SD, standard deviation. Post-secondary defined as at least “university or other post-secondary education, less than 4 years”. Moderate/high activity defined as at least “≥3 h light activity/week or <1 h vigorous activity/week”. | | | | | | | | | | |
|  |  |  |  |  |  |  |  |  |  |  |
