# Supplementary material for: Body mass index and risk of dying from a bloodstream infection: A Mendelian randomization study
Source: PLoS Med. 2020 Nov 16;17(11):e1003413. doi: 10.1371/journal.pmed.1003413 (PMC7668585; doi:10.1371/journal.pmed.1003413)
Supplement: S5 Table — HR, hazard ratio; IVW, inverse-variance-weighted; OR, odds ratio. The analyses assume a linear relationship between body mass index and bloodstream infection mortality in the general population using the same 939 single nucleotide polymorphisms (SNPs) as used to create the genetic risk score. Two-sample analyses use SNP–exposure associations from Yengo et al. [29] and SNP–outcome associations from HUNT. The I2 values of the SNP–exposure associations were 54% in the 1-sample MR-Egger regression and 92% in the 2-sample MR-Egger regression. Effect estimates reported as HR for 1 unit increase of body mass index in 1-sample analyses and as OR for 1 standard deviation increase of body mass index in 2-sample analyses. (DOCX) [file pmed.1003413.s014.docx]

| **S5 Table. Mendelian randomization sensitivity analyses of linear association between body mass index and bloodstream infection mortality in the general population** | | | | | | | | | |
| --- | --- | --- | --- | --- | --- | --- | --- | --- | --- |
|  | HR/OR | Lower | Upper | P-value |  | Intercept | Lower | Upper | P-value |
| **One-sample** |  |  |  |  |  |  |  |  |  |
| MR-Egger, random effects | 1.18 | 1.04 | 1.33 | 0.011 |  | 1.00 | 0.99 | 1.00 | 0.476 |
| IVW, random effects | 1.13 | 1.05 | 1.23 | 0.002 |  | - | - | - | - |
| Median estimator, weighted | 1.13 | 0.99 | 1.30 | 0.081 |  | - | - | - | - |
| **Two-sample** |  |  |  |  |  |  |  |  |  |
| MR-Egger, random effects | 1.98 | 0.95 | 4.18 | 0.070 |  | 1.00 | 0.99 | 1.01 | 0.877 |
| IVW, random effects | 1.89 | 1.33 | 2.67 | <0.001 |  |  |  |  |  |
| Median estimator, weighted | 2.09 | 1.10 | 3.97 | 0.025 |  |  |  |  |  |
| HR, hazard ratio; IVW, inverse-variance weighted; OR, odds ratio. Assuming a linear relationship between body mass index and bloodstream infection mortality in the general population using the same 939 single nucleotide polymorphisms (SNP) as used to create the genetic risk score. Two-sample analyses use SNP-exposure associations from Yengo et al [ref 2 in Supplementary text], and SNP-outcome associations from HUNT. The I^2^ of the SNP-exposure associations were 54% in the one-sample MR-Egger regression, and 92% in the two-sample MR-Egger regression. Effect estimates reported as HR for one unit increase of body mass index in one-sample analyses and as OR for one standard deviation increase of body mass index in two-sample analyses. | | | | | | | | | |
