# Supplementary material for: Body mass index and risk of dying from a bloodstream infection: A Mendelian randomization study
Source: PLoS Med. 2020 Nov 16;17(11):e1003413. doi: 10.1371/journal.pmed.1003413 (PMC7668585; doi:10.1371/journal.pmed.1003413)
Supplement: S6 Table — BMI, body mass index; BSI, bloodstream infection; Q, quartile; SD, standard deviation. The final column, for dichotomous covariates, compares the Q4/Q1 ratio among patients with BSI with the Q4/Q1 ratio in the general population (S3 Table), and similarly compares the mean difference of Q4 − Q1 among BSI participants with Q4 − Q1 in the general population. Post-secondary defined as at least some university or other post-secondary education. Moderate/high activity defined as ≥3 h light activity/week or any vigorous activity/week. (DOCX) [file pmed.1003413.s015.docx]

| **S6 Table. Distribution of potential confounders by body mass index quartiles among patients with bloodstream infection** | | | | | | | | | | | | |
| --- | --- | --- | --- | --- | --- | --- | --- | --- | --- | --- | --- | --- |
|  | **Quartiles of BMI** | | | | | | | | | |  |  |
|  | **Q1** | | **Q2** | | **Q3** | | **Q4** | | **Test for trend** | |  | **Difference between  BSI-patients and total population** |
| Quartile range, kg/m^2^ | 14.8 to 23.5 | | 23.6 to 25.8 | | 25.9 to 28.6 | | 28.7 to 53.3 | |  |  |  |  |
|  |  |  |  |  |  |  |  |  |  |  |  |  |
|  | *n* | *%* | *n* | *%* | *n* | *%* | *n* | *%* | *R2* | *P-value* |  | *Difference in proportion of Q4/Q1 (%-points)* |
| Male sex | 192 | 46.8 | 314 | 59.9 | 416 | 60.5 | 422 | 45.7 | 0.1 | 0.048 |  | -28.1 |
| Self-reported cancer | 34 | 8.9 | 28 | 5.9 | 31 | 4.9 | 51 | 6.2 | 0.2 | 0.136 |  | -68.0 |
| Never smoked | 120 | 30.1 | 165 | 32.0 | 224 | 33.3 | 367 | 40.6 | 0.5 | <0.001 |  | 31.1 |
| Current smoker | 184 | 46.1 | 169 | 32.8 | 180 | 26.7 | 190 | 21.0 | 2.8 | <0.001 |  | -15.1 |
| Post-secondary education | 49 | 12.9 | 78 | 16.1 | 67 | 10.6 | 80 | 9.5 | 0.5 | 0.004 |  | 16.6 |
| Moderate/high activity | 172 | 52.3 | 256 | 60.0 | 315 | 56.8 | 347 | 47.2 | 0.3 | 0.007 |  | 9.7 |
|  |  |  |  |  |  |  |  |  |  |  |  |  |
|  | *Mean* | *SD* | *Mean* | *SD* | *Mean* | *SD* | *Mean* | *SD* |  |  |  | *Difference in means of Q4-Q1* |
| Age | 58.0 | 15.5 | 61.7 | 13.2 | 63.3 | 12.4 | 62.1 | 12.6 | 0.8 | <0.001 |  | -5.2 |
| BMI | 21.7 | 1.5 | 24.8 | 0.7 | 27.2 | 0.8 | 32.3 | 3.5 | 72.8 | <0.001 |  | 0.5 |
| BMI, body mass index; BSI, bloodstream infection; Q, quartile; SD, standard deviation. The final column, for dichotomous covariates, compares the Q4/Q1-ratio among patients with BSI with the Q4/Q1-ratio in the general population (Supplementary Table 2A), and similarly compares the mean difference of Q4-Q1 among BSI-subjects with Q4-Q1 of general population. Post-secondary defined as at least “university or other post-secondary education, less than 4 years”. Moderate/high activity defined as at least “≥3 h light activity/week or <1 h vigorous activity/week”. | | | | | | | | | | | | |
