# Supplementary material for: Body mass index and risk of dying from a bloodstream infection: A Mendelian randomization study
Source: PLoS Med. 2020 Nov 16;17(11):e1003413. doi: 10.1371/journal.pmed.1003413 (PMC7668585; doi:10.1371/journal.pmed.1003413)
Supplement: S1 Text — (DOCX) [file pmed.1003413.s016.docx]

**S3 TEXT - SUPPLEMENTARY DESCRIPTION OF METHODS**

**Brief description of fractional-polynomial method**

Based on residual body mass index (BMI) values (i.e. after subtracting the genetic component of BMI), we divided the population into 20 equally sized strata (30 for bloodstream infection (BSI) incidence), and within each stratum we calculated the ratio between the GRS–outcome association, and the GRS–exposure association. We then performed meta-regression of these estimates against the mean of BMI in each stratum in a fractional polynomial model of degrees 1 and 2. Stratifying on residual BMI avoids introducing additional collider bias as – unlike uncorrected BMI – residual BMI is not on the causal pathway from the genetic variants to the outcome.

**Genotyping and imputation**

DNA was genotyped using one of three different Illumina HumanCoreExome arrays (HumanCoreExome12 v1.0, HumanCoreExome12 v1.1 and UM HUNT Biobank v1.0). Samples were excluded if they had a call rate <99%, had large chromosomal copy number variants, contamination >2.5% as estimated with BAF Regress (1), or discordance between genotypic and phenotypic sex. Samples not of recent European ancestry were excluded. Genetic variants were excluded if they were out of Hardy-Weinberg equilibrium (p-value <0.0001), or call rate <99%. Imputation was thereafter performed using Minimac3 using 2201 whole-genome sequences from HUNT and HRC v1.1.

**Two-sample Mendelian randomization analyses**

In sensitivity analyses, we conducted two-sample Mendelian randomization (MR) analyses of the main outcome (association between BMI and risk of dying from a BSI in the general population) for comparison with our one-sample estimates. For BMI, we used the same instruments from Yengo et al as used to create the genetic risk score (2). SNP-outcome estimates were calculated by running SAIGE (v 0.29.4) with age, sex, and five first ancestry-informative principal components as covariates (3). Cases were defined as subjects who died from a BSI, while controls were defined as subjects followed for at least 8 years and who had no hospitalizations with infections. MR-Egger regression, inverse-variance weighted regression, and weighted median estimator were run as described in the methods section in the paper.

**References**

1. Jun G, Flickinger M, Hetrick KN, Romm JM, Doheny KF, Abecasis GR, et al. Detecting and estimating contamination of human DNA samples in sequencing and array-based genotype data. Am J Hum Genet. 2012;91(5):839–48.

2. Yengo L, Sidorenko J, Kemper KE, Zheng Z, Wood AR, Weedon MN, et al. Meta-analysis of genome-wide association studies for height and body mass index in ∼700000 individuals of European ancestry. Hum Mol Genet. 2018 Oct 15;27(20):3641–9.

3. Zhou W, Nielsen JB, Fritsche LG, Dey R, Gabrielsen ME, Wolford BN, et al. Efficiently controlling for case-control imbalance and sample relatedness in large-scale genetic association studies. Nat Genet. 2018 Sep 13;50(9):1335–41.
