# Supplementary material for: Body mass index and risk of dying from a bloodstream infection: A Mendelian randomization study
Source: PLoS Med. 2020 Nov 16;17(11):e1003413. doi: 10.1371/journal.pmed.1003413 (PMC7668585; doi:10.1371/journal.pmed.1003413)
Supplement: Attachment — Submitted filename: Response_to_comments_revision3.docx [file pmed.1003413.s018.docx]

**Oct 1^st^ 2020**

**EDITOR'S REQUESTS/COMMENTS:**

1. Thank you for editing the data availability statement. I believe that it only needs to be in the metadata section and not the main text, but defer to the production team on this point.

*Response:*

*Yes, the production team brought this up as well, and the text has now been limited to the metadata section.*

2. Thank you for including p-values. However please report only down to three decimal places, and as p< 0.001 if lower. The exception is if it is statistically relevant to report the lower values.

*Response:*

*Thanks, this has now been revised.*

3. Please include your response to reviewer 4 (regarding the bias due to the binary outcome not due to confounding) in the main text, in case other readers have the same question.

*Response:*

*Thanks, this has been added to the discussion, lines 424-427.*
