# Supplementary material for: Body mass index and risk of dying from a bloodstream infection: A Mendelian randomization study
Source: PLoS Med. 2020 Nov 16;17(11):e1003413. doi: 10.1371/journal.pmed.1003413 (PMC7668585; doi:10.1371/journal.pmed.1003413)
Supplement: Attachment — Submitted filename: Response to reviewer comments.docx [file pmed.1003413.s017.docx]

**Comments from editors and reviewers**

**Requests from the editors:**

*Please structure your abstract using the PLOS Medicine headings (Background, Methods and Findings, Conclusions) - the Methods and Findings section should be one single section (“Methods and findings”).

*Response:*

*The abstract has been reformatted accordingly (page 7).*

*In the last sentence of the Abstract Methods and Findings section, please describe the main limitation(s) of the study's methodology.

*Response:*

*This has been done (lines 126-128)*

*At this stage, we ask that you include a short, non-technical Author Summary of your research to make findings accessible to a wide audience that includes both scientists and non-scientists. The Author Summary should immediately follow the Abstract in your revised manuscript. This text is subject to editorial change and should be distinct from the scientific abstract. Please see our author guidelines for more information: <https://journals.plos.org/plosmedicine/s/revising-your-manuscript#loc-author-summary>

*Response:*

*A non-technical summary has been added, following the abstract (pages 8-9).*

*Although in general the paper is very clearly reported, the authors might wish to know that there is a draft guideline, STROBE-MR, for reporting Mendelian Randomization studies (<https://peerj.com/preprints/27857/>) - and they may wish to use, and reference, this guideline to help enhance the depth of reporting in their paper, if appropriate.

*Response:*

*We greatly appreciate the effort to standardize the reporting of MR-studies, and we have therefore followed the STROBE-MR checklist, and cited the pre-print in the beginning of the methods section (lines 218-219).*

**Reviewer #1:**

This is an interesting study assessing the causal relationship between body mass index and bloodstream infection (risk and mortality) under a Mendelian Randomization framework. The authors used information on 55,908 participants of the HUNT study that were followed during a median of 21 years. In order to allow for non-linear associations, they also conducted a non-linear Mendelian randomization approach. They found that elevated genetically-predicted BMI was causally associated with increased risk and mortality of bloodstream infection. 
Given that this is the first study evaluating the causal association between body mass index and bloodstream infection in a Mendelian randomization framework, in addition to allowing for non-linear associations, these are interesting results that can help to understand the mechanisms of body mass index on health outcomes. 
I found the article well organized and clearly written. The main and sensitivity statistical analyses are appropriate, and the results and conclusions are generally well presented.
I only have two minor comments regarding the presentation of the results.
1.	As in Supplemental Table 1, the authors should add a table with the results for the association between BMI and bloodstream incidence. These results could also be added in Supplemental Table 1 in new rows.

*Response:*

*We thank the reviewer for the thorough review of our manuscript. The suggested table has been added. Supplementary Table 1 is now a sensitivity analysis for BSI incidence, and the previous Supplementary Table 1 has been changed to Supplementary Table 2. The BMI-stratum sensitivity analyses of BSI incidence observe the same thing as the comparable analysis for BSI mortality in the general population: The traditional multivariate analyses are slightly closer to the null compared with the MR analyses, which suggests that there is some negative residual confounding in the traditional analyses.*

*While the supplementary analysis has been added in the revised manuscript, we still think it would be better if it is left out. The reason for this is two-fold. First, all other sensitivity analyses are based on the main association of BMI and BSI mortality in the general population; the new Supplementary Table 1 deviates from this as it explores another association. And second, the manuscript is already dense with sensitivity analyses, and we are concerned that adding more sensitivity analyses may reduce readability.*

2.	In "Supplemental Table 3: Evaluation of MR-assumptions" the title is not clear. Are those the results from the robust MR methods (MR-Egger, IVW and weighted median) for the association between BMI and bloodstream infection mortality? Or incidence? Or it is another thing? Please clarify. In fact, it would be good to have a table with the overall results for bloodstream infection mortality and incidence with the distinct techniques used in this study: 2 stage least squares, MR-Egger, IVW, weighted median, etc. This table would strengthen the presentation of the results.

*Response:*

*We agree with the reviewer that Supplemental Table 3 was difficult to interpret in its previous form. We have now created a title that properly declares what is presented in the table. The footnotes have also been made more descriptive. The table has been expanded to include two-sample sensitivity analyses (see Reviewer #4, comment #5). Finally, as another table has been added above, the table is now Supplementary Table 4.*

*While we understand the reviewer’s point in presenting all findings in one table, we are not fully convinced that this will benefit the paper. Our goal is that in a formatted paper, the three main figures (Figures 1, 2 and 3) will convey the main message, and will be easiest to follow. All the sensitivity analyses (e.g. MR-Egger) are simply analyses to evaluate the robustness of the overall message, and, we argue, should not be presented as ‘main results’. All sensitivity analyses are based on the main analysis; genetically-predicted BMI on risk of dying from a BSI in the general population. To properly make the three main figures stand out, we have decided to move the sex-stratified analyses of bloodstream infection (BSI) mortality in the general population to the online supplement.*

Suggestion: I would combine Figure 4A and Figure 4B in a single figure with 2 panels. The same for Supplemental Figure 1A and 1B, and for Supplemental Figure 4A and 4B. By showing together the associations for men and women (never smoker and ever smoker) you will help the reader to see the comparisons more easily.

*Response:*

*This is a great idea, and what we had hoped to achieve at the formatting stage of publication. These are all supplementary figures, and we have now create two panels for each of these figures.*

-----------------------------------------------------------

**Reviewer #2:**

In this manuscript, authors use a population-based cohort from Norway with 23 years follow up and evaluated the impact of BMI on the risk of bloodstream infection and associated mortality risk. They use Mendelian randomization in an attempt to minimize bias, confounding and reverse causation. I agree that associations in observational studies are subject to a number of biases and attempts to obtain answers using methodologies that mitigate bias, especially for clinically important questions such as this are warranted.  However, I have a number of concerns that I think need to be addressed first. 

Major

1. It is unclear to me as to whether authors correlated genotype-predicted BMI with actual BMI. Please explain. Granted that the genetic predisposition for higher BMI is a nice plus to this study, however BMI is also very much impacted by environmental factors and as such, BMI changes considerably in sepsis/bacteremic patients on presentation especially when they receive significant fluid resuscitation. The time interval between BMI measurement and onset of BSI is very relevant. It is difficult to associate BMI with outcome if the two are very far apart in time. Please compare results of predicted BMI and actual BMI based associations.

*Response:*

*We greatly appreciate the reviewer raising these issues, as they may be a source of confusion to many readers. Apart from the sensitivity analyses where we explicitly say that we evaluate the association between observed BMI (as opposed to genetically-predicted BMI) and BSI mortality (previously Supplementary Table 1, now Supplementary Table 2), we only evaluate the effect of genetically-predicted BMI. In the revised manuscript, we have made it clearer that we are considering the effect of genetically-predicted BMI throughout, except where otherwise stated (including explicitly mentioning this in lines 266-267).*

*The reviewer is right that we have not explicitly stated the correlation between observed BMI and genetically predicted BMI. However, this is the same as the correlation between the genetic risk score used to calculate the genetically predicted BMI, and observed BMI. As stated in the second paragraph of the results section, the genetic risk score for BMI explains 4.2% of the variation in observed BMI; thus, genetically-predicted BMI also explains 4.2% of the variation of observed BMI. We are using genetic variation as instruments for BMI. An MR-analysis is comparable to an intention-to-treat analysis in an RCT, where subjects are defined based on treatment allocation, and not actual treatment: Low adherence to treatment allocation (genetic predisposition) will reduce statistical power, but will not otherwise bias the results, assuming that lack of adherence is random. In other words, even though the genetic risk score ‘only’ explains 4.2% of the variation in BMI, this is principally a matter of statistical power, and was overcome by using a large study population.*

*The reviewer raises some important points regarding the environmental influence on BMI and the time interval between measured BMI and hospitalization with BSI. While observed BMI will necessarily be affected by BSI status (e.g. dehydration or fluid resuscitation), this will not be the case for genetically-predicted BMI, which is assigned at conception. Indeed, this is one of the advantages of using genetically-predicted BMI, as it will, among other things, not be affected by reverse causation or confounding. The same argument holds for the time interval between ascertainment of BMI and hospitalization with BSI: This bias was avoided in all our analyses (save the sensitivity analyses where observed BMI was used) as we use genetically-predicted BMI.*

*Finally, the reviewer asks for a comparison between the effect of observed BMI on BSI risk/mortality with that of genetically-predicted BMI on BSI risk/mortality. In terms of risk of dying from BSI in the general population, this comparison is available in Supplementary Table 2 (previously Supplementary Table 1). As can be appreciated, the findings are in the same direction using both techniques, but the harmful effect of increasing BMI is more pronounced in the MR analyses (which suggests that there may be some additional negative confounders not accounted for in the traditional multivariate analyses). With the revisions, we have also added a supplementary table on risk of contracting BSI comparing MR and traditional analyses (Supplementary Table 1), where we again find that the traditional multivariate analyses are slightly closer to the null.*

2. Authors had access to bloodstream pathogen species (to be able to exclude contaminant species). Please provide sensitivity analyses by organism type (gram positive ve negative), It would be valuable to know of there is pathogen-level heterogeneity of outcome.

*Response:*

*It is a good suggestion to include sensitivity analyses on gram-positive vs gram-negative BSI. We have conducted a stratified sensitivity analysis of the main outcome, i.e. an MR analysis of BSI mortality in the general population separately for gram-positive and gram-negative BSIs (Supplementary Figure 7). It is interesting to note that for the lower levels of BMI (<25 kg/m^2^) there is no strong positive association between genetically-predicted BMI and BSI mortality among gram-negative BSIs; indeed, there is a tendency of a protective effect, but with very wide confidence intervals. Among the overweight and obese, the associations are similar for gram-positive and gram-negative BSIs.	These new analyses are described in the methods (lines 313-314) and results section (lines 409-412). As a discussion of gram-positive vs gram-negative infections is outside the main and secondary objectives of the current work, we have not elaborated on the slightly discrepant results between gram-negative and gram-positive BSIs.*

3. The first sentence of the abstract is long and confusing as written. Please have succinct statements in the abstract so you do not lose your audience at the outset.

*Response:*

*We agree with the author that this sentence was long-winded, and we have split it into two sentences with a clearer message (lines 110-113).*

4. Some more detail on the way patients were followed up would be helpful. How often were they seem after the initial survey? How often were measurements llke BMI done.

*Response:*

*In HUNT2, the study participants had one or more visits where lifestyle factors, measurements, and blood samples were collected, but they were not ‘followed’ over time with repeated visits in the HUNT2 survey. BMI was only recorded once in HUNT2 for each participant. We were able to reconstruct follow-up by doing record linkage with all the hospitals in the area (for hospitalizations), and through linkage with the National Registry (date of emigration out of the county, and date of death). Thus, we were able to successfully account for time at risk.*

*Given that our exposure of interest is genetically-predicted BMI, the frequency of the follow-up is not of great importance. In an MR model, it is usually not necessary to include covariates in the model to adjust for confounding (ancestry-informative principal components may be included to reduce risk of confounding from population stratification in cohorts with diverse ancestry composition), but age and sex are often added to improve statistical power. Thus, the quality of the information collected from surveys (e.g. smoking habits) are of less importance in an MR study compared with a traditional epidemiological study.*

*That being said, we used non-genetic information gathered from the HUNT2 survey to inform our inverse-probability weighted analyses of BSI case fatality. In this analysis, we estimated the probability of developing a BSI given the information gathered at HUNT2 participation, and assigned more weight to those with the lowest risk. For this analysis to be valid, it is important that information is gathered similarly for all subjects, which it was, but the analyses should not otherwise be affected by the frequency of information ascertainment.*

*We have added a phrase in the methods section underscoring that information was gathered ‘once for each participant in the HUNT2 survey’ (line 228).*

5. Authors appear to have aces to microbiology data from the hospitalizations at 2 community and 1 tertiary care hospital. WHat other variables did they have access to among hospitalized patients?

*Response:*

*See response below.*

6. Related to point #5, I think one of the biggest limitations of this study is the lack of physiologic and lab variables for risk adjustment of severity of acute illness. These data might be available from the hospital encounters. In line with authors attempts to minimize confounding, the elephant in the room is the absence of adjustment for baseline acute illness upon presentation with BSI. This really biases the outcome and put authors' findings and their claims into question.

*Response:*

*This is yet another important point, and we thank the reviewer for bringing it up. Currently, diagnosis codes at discharge are available for linkage, but we have not attempted to do such linkage for this work. Unfortunately, we do not have information on laboratory results, clinical examination etc. We agree with the reviewer that it would be nice to have this information to more properly describe the BSI patient population. On the other hand, we are of a different opinion when it comes to the validity of our findings. As we have described previously in this document as well as in the body of the manuscript, the use of genetic instruments will reduce the risk of confounding without the need for including potential confounders in the statistical model. Furthermore, many baseline characteristics at hospitalization will not be confounders of the BMI-BSI-mortality association, but mediators. Imbalance of baseline characteristics at hospitalization may also be a result of selection bias, and this potential bias is not correctly dealt with by including these characteristics as covariates in a model* [1]*. Thus, even if we had information on lab values and clinical characteristics, this information would not have been included in our MR models as our MR models are more valid as they are currently presented.*

7. The discussions section on "Strengths and Limitations" mostly displays strengths alone. Please report limitations as well.

*Response:*

*We have expanded the strengths and limitations section and highlighted the potential risk of pleiotropic effects, along with reduced power due to the low sensitivity of blood culture tests.*

8. Authors need a stronger conclusion statement. Saying that this work promote efforts to encourage weight loss is not enough. 

*Response:*

*We have added a sentence where we state that our findings do not support research into how a high BMI is protective in terms of mortality from infectious diseases (lines 486-513).*

Minor 

1. Lack of line numbers makes the review process a bit more cumbersome.  

*Response:*

*We agree that line numbers make the review process easier, and have now added that in the revised manuscript.*

2. On page 11, paragraph 2: what was the basis of choose 30 days? Any justification for the same?

*Response:*

*We pragmatically decided that death within 30 days of BSI should be attributed to death due to BSI. Some authors choose a slightly different time interval, e.g. 28 days, but the findings would be the same.*

-----------------------------------------------------------

**Reviewer #3:**

Thank you for asking me to review this article which concerns the relationship between obesity and blood stream infection (BSI). There remains considerable controversy over the relationship between sepsis mortality and obesity, with conflicting results from studies which have used a variety of methods to investigate the relationship.  In this manuscript Rogne and colleagues use a Mendelian randomisation technique to examine the relationship between genetically predicted body-mass index and BSI incidence and mortality, demonstrating positive relationships between both these and genetically predicted BMI.  As the first MR-based study to investigate the relationship between genetically predicted obesity and BSI incidence/mortality, this is a significant addition to the literature which overcomes several of the problems associated with conventional observational studies.   As the authors correctly note, BSI and sepsis are not synonymous although most patients with
BSI will meet the current criteria for sepsis and therefore these findings are likely to be of relevance beyond BSI and indicate methods for similar studies in other infectious disease syndromes.

This is a well conducted study with a rigorous approach to examining potential confounders, and a measured discussion of the findings with due note given to findings such as the potential J-shaped relationship without over-interpretation.  My only comment is that although positive blood cultures (excluding common contaminants) is specific for blood stream infection, it is insensitive as it relies on the growth of bacterial organisms - which either organism fastidiousness or inter-current antibiotics may prevent.   It is therefore possible, indeed likely, that some of the individual included in this study as 'non-BSI' patients will have had a missed BSI.  Recent studies of molecular diagnostics, (e.g. Nguyen MH, et al. Annals of Internal Medicine 2019) suggest that BSI may be over 4 times more prevalent amongst hospitalised patients than is detected by growth-based methods.  I suggest that the authors discuss this as a potential weakness in this study, and make it clear that
their findings  -at present- relate only to culture-positive BSI.

*Response:*

*We thank the reviewer for considering our work, and for addressing a concern we share. Although the specificity of blood cultures is excellent, the sensitivity leaves much to be desired. We therefore suspect that we may have missed several BSI cases. This contribution to noise and lack of case identification will tend to make the analyses more conservative, which we have overcome with the large sample size. We have added a succinct discussion of this in the limitations section of the revised manuscript (lines 508-511).*

*The reviewer noted that this is the first study to evaluate the association between BMI and BSI/sepsis in an MR setting, which it was at the time of submission. Although it does not affect the impact of the present work, we want to notify the editors that while our manuscript was under peer-review, a paper was published that has a slight overlap in scope with the current manuscript* [2]*. In that paper, the authors evaluated the association between BMI and risk of several infectious diseases – including sepsis – among ~100,000 Danes. In traditional (i.e. non-MR) analyses, they found that BMI was positively associated with risk of sepsis, similarly to what we found. In their MR-analyses, however, they found no association between BMI and risk of developing sepsis. We speculate that this lack of association in the MR-setting was because they used a very weak instrument of only 5 SNPs (as they did not have genome-wide genotyping), and they had fewer cases than in our work. Importantly, they did not evaluate the risk of death due to sepsis, either in the total population, nor among sepsis patients. Thus, they did not address the obesity paradox, which is one of the most important contributions of our work. We are therefore confident that the current manuscript is still of considerable interest to your readership, as we are the first to demonstrate an unconfounded association between BMI and risk of BSI (and likely sepsis), BSI mortality in the general population, and BSI case fatality. We have rephrased the manuscript throughout so that we do not erroneously state that ours is the first study to use an MR design to assess the association between BMI and risk of sepsis (lines 487-488).*

-----------------------------------------------------------

**Reviewer #4:**

Body mass index (BMI) has been associated with the risk of mortality of bloodstream infection (BSI). But the direction of the effect is unclear, because BMI seems to be a risk factor in the general population and a protective factor in patience with BSI and sepsi.
The authors performed a one-sample Mendelian randomization (MR) analysis using the HUNT study. MR analyses were conducted with a weighted genetic risk score composed of 939 independent variants. That study support the hypothesis of BMI as a risk factor.

The paper is well written. The MR study is very well performed. My specific comments are detailed hereafter.
1.	The authors should comment about the bias due to the binary outcome

*Response:*

*As instrumental variable analyses do not adjust for covariates, estimates are marginal across covariates. This differs from estimates from multivariable-adjusted regression, which are conditional in covariates that are included in the regression model. We have discussed this in the discussion section (lines 441-445)*

2.	However, as a strength they should also mention that those analyses are not biased by the winner course.

*Response:*

*We thank the reviewer for reminding us of this. Indeed, given that the instruments were selected from an independent sample, our MR analyses will tend to be conservative. We have included a sentence about this in the strengths and limitations section (lines 497-498).*

3.	About the MR-Egger, they should comment something about the measurement error.

*Response:*

*We have included the I^2^ GX statistic from the MR-Egger analyses in Supplementary Table 4 (previously #3), which is a measure of the attenuation due to measurement error in the gene—risk factor associations. In the one-sample analysis, the I^2^ was 54%, which is not ideal, and part of the reason why we wanted to evaluate MR-Egger regression analyses in a two-sample design, see below. In the latter analysis the I^2^ was 92% and the NO Measurement Error assumption of MR-Egger may be assumed to hold* [3]*; the analysis was comparable (more pronounced effect) to the one-sample analysis.*

4.	In my opinion, in order to evaluate the robustness of sensitivity analyses, they should comment about additional assumptions, which are made for each MR method. For example, what about the independence between GX and GY associations in the context of one-sample MR study.

*Response:*

*If the gene—exposure and gene—outcome associations are estimated in the same individuals (one-sample analysis), then there will be some bias in the MR estimates towards the observational association. However, if the gene—exposure associations are strong, then bias is not substantial. In our investigation, the F statistic from regression of the exposure on the genetic variants was 2461, suggesting that bias in not substantial. Please also see the response to comment #5.*

5.	They should comment on the use of two-sample MR methods using one sample. Maybe, it could be interesting to compare their results with a MR study performed using GX and GY estimates from two different data sources, which have to be homogeneous, of course.

*Response:*

*Use of two-sample methods in a one-sample setting is feasible, but – as the reviewer correctly points to – comes with some limitations. Principally, calculation of Wald ratios for each instrument, and then summarizing across these instruments to estimate an overall effect, is similarly done in both two-sample and one-sample settings. A valid instrument should be associated with the outcome only through the exposure. The risk of violating this assumption is greater in a one-sample setting given that both GX and GY are estimated from the same population. In particular, there is a risk that the genetic instruments are associated with one or more confounding factors, such as smoking, which in turn may affect both BMI and risk/mortality of BSI. Thus, the one-sample estimate will have a greater tendency to bias towards the confounded estimate than a two-sample estimate. This will be the case for the IVW regression, and to a smaller extent of MR-Egger regression. The weighted median estimator will still be correct, given that at least half the instruments are valid: As mentioned, the risk is higher in a one-sample than two-sample design that more instruments violate the MR assumptions, hence the weighted median estimator will also be more likely to be biased towards the confounded estimate.*

*As suggested by the reviewer, we have conducted two-sample MR sensitivity analyses of the main outcome, BSI mortality in the general population, using the association between genetic instruments and BMI from the 700,000 sample in the Yengo et al paper, and between the same instruments and BSI mortality in the HUNT population. The methods are described in the online supplement. In these analyses, we find a more pronounced association between BMI and BSI mortality than we did in the one-sample analyses, but with greater standard errors. These findings suggest that if there is any residual confounding in the one-sample analyses, it will likely bias the presented estimates towards the null (e.g. negative confounding from smoking which reduces BMI but increases mortality risk). The larger standard errors suggest heterogeneity between the populations from which the SNP-exposure and SNP-outcome associations were drawn. We have included the results from this sensitivity analysis in Supplementary Table 3 and lines 396-400, along with describing the methods briefly in the manuscript (lines 299-302) and more elaborately in the online supplement, and we have added a few sentences to the discussion (lines 479-484).*

6.	Just a small comment, be consistent with the notation for confidence intervals, don't use the dash after 95% CI, but for example the colon

*Response:*

*We apologize for the inconsistent notation, and have revised our manuscript to use ‘95% CI:’ throughout (except in the abstract).*

**References**

1.	Banack HR, Kaufman JS. From bad to worse: collider stratification amplifies confounding bias in the “obesity paradox.” Eur J Epidemiol **2015**; 30:1111–1114.

2.	Winter-Jensen M, Afzal S, Jess T, Nordestgaard BG, Allin KH. Body mass index and risk of infections: a Mendelian randomization study of 101,447 individuals. Eur J Epidemiol **2020**;

3.	Bowden J, Fabiola Del Greco M, Minelli C, Smith GD, Sheehan NA, Thompson JR. Assessing the suitability of summary data for two-sample mendelian randomization analyses using MR-Egger regression: The role of the I2statistic. Int J Epidemiol **2016**; 45:1961–1974.
